# Indents

**Published:** 1920-03

**Authors:** 


### INDENTS

**☞Brief Articles of Technical or Scientific Value will receive notice and comment. Contributions invited.**

---

Clasps. After experimenting with a number of different kinds of attachments and clasps, I have eliminated them all except three: the flat plate clasp, the half-round wire clasp and the cast clasp. The last named has a very limited field. It should only be used on the small Bonwill type of bridges. In many instances, even in this type of work, the half-round wire clasp could be made in less time, give just as satisfactory results, and would probably be stronger and better conformed to the principles of clasp construction that have been laid down by Dr. Bonwill. He made it clearly understood thirty years ago that the clasp should cover the greatest diameter or bulge of the tooth, should have a resting lug on the occlusal surface at a point which corresponds to the contact point the natural tooth would have. This is done to prevent the appliance from being forced toward the gum, and also to prevent portions of food from packing between the clasps and the teeth which would ultimately force the gum back at this point, forming a pocket and causing subsequent loss of this tooth. Half-round wire clasps are used where the teeth are very short and the greatest diameter is close to the gum. If the appliance settles, this type of clasp more readily allows for adjustment. It is also used in the anterior part of the mouth where it is desirable to show as little gold as possible.

There have been many arguments pro and con in the use of the clasp. The objection has been raised that they wear off the enamel by actual abrasion. I do not believe this to be the case. On the other hand, I have seen numerous instances where the clasp has been worn through by the friction of the teeth themselves with no appreciable wear to the enamel. I have found if there is any wear it is not the abrasive action of the clasps (for if you stop to think, we know that the clasp is of a softer material than the enamel itself), but is due to the collection of a film of partly disintegrated food which is left on the inside of the clasp and eventually dissolves the substance of the enamel which renders the tooth softer than the clasp and then the enamel is rubbed away by the movement of the clasp. If the patient gives the clasp the necessary care, this objection can be entirely overcome, and I can say that in the approximately four years that I have been keeping a record of the cases where the enamel has disintegrated under the action of the clasps, I find that I have only two such cases. In both instances the patients' care of their plates was far from satisfactory. I think it is a good thing to have the clasp absolutely polished on the inside and the patient should be instructed to polish the clasps once a day with an orange-stick and bicarbonate of soda. It takes only a moment to do this work, and the clasps will wear well and give good results. I have seen a number of good teeth covered with crowns, the dentist explaining to the patient that they would prevent the teeth from wearing out. If he had stopped and thought that to properly make a crown for a tooth it would necessitate so much cutting as to need devitalization, he would have placed the clasp directly on the tooth. If decay occurred after a number of years he would have recourse to a filling and finally to a porcelain jacket crown. At any rate the life of the pulp would have been conserved.

In no case should an extension bar be placed back of the tooth, as it will act as a lever and the tooth will be dragged out of position, resulting in its loss. We should carefully study the position of the teeth we are clasping and select teeth which are parallel with each other. If we do not do so, constant putting in and taking out of the plate will very soon loosen the teeth. We should look to the occlusion and so place the teeth on the plate as to prevent rocking, not depending on the clasp to do this, for if we do the teeth they are attached to will ultimately be loosened and lost. The general principle of the clasp should only be used to prevent the plate from moving when excessive motion of the mouth is made in mastication and speech.

I have neglected to mention one form of clasp which I have used very successfully in the type of cases where the teeth are lost on one side of the mouth, but on the opposite side are perfect, with no fillings or crowns. In these cases I have used the "Jackson Crib." It was primarily designed for use as a regulating appliance, but works admirably in the cases just referred to where we do not care to devitalize or cut the teeth.—Edward Kennedy, in *Cosmos.*

---

Caries and Mouth Breathing. Referring to a letter in the *British Medical Journal* from Dr. W. H. Brown, noting that dental decay sometimes follows on mouth breathing, another writer (Capt. C. J. Hill Aitken) in that journal mentions the case of an officer of low category who was a mouth breather. By carrying out the advice given he become a nasal breather, and was transformed from semi-invalidism to health. Seen eighteen months later, there was clear evidence of arrested decay in three teeth that had large cavities. The officer remarked: "I was never out of the dentist's hands from my boyhood until the time I had the luck to meet you and got your tip. I have made no change in my life except I breathe through my nose."—*Dental Record.*

---

Too Many Doctors. A dim warning of the danger of multiplying the number of practitioners beyond that required to meet the effective demand may be read (by the timid ones) into the following report taken from a contemporary:

"There are in Spain, a country of twenty million inhabitants, ten schools of medicine, and the number of students exceed those enrolled in the other subjects (law, pharmacy, science and others). All cities and villages are overcrowded with physicians. As the supply exceeds the demand by far, the medical service is degraded. Some physicians charge as low as two cents per visit and treat patients at the monthly rate of one peseta (nineteen cents). There are over two thousand physicians in Madrid, a city of 600,000 inhabitants. The real trouble is that physicians—instead of helping themselves—want some one to help them out and place all their hopes in the government.''—*Dental Record.*

---

Treatment of Acute Septic Gingivitis (Vincent's Disease). As one sees nowadays so many cases of this disease, I should like to suggest a simple treatment which I have found most effective. After the usual routine treatment of antiseptic mouthwash, iodin, scaling and removal of septic roots, I instruct the patient to have the gums syringed two or three times a day with potassium permanganate solution (1:5000); ''the color of pink blotting paper'' is quite enough guide as to the strength of the solution. A four-ounce glass or an ear syringe with a fine nozzle may be used, the essential part of the syringing being to use a good deal of force. Have the syringe about one foot away from the patient's mouth, and freely syringe all affected parts; this washes away the pus, sloughs and grayish membrane from the gums and between the teeth. The patient will always find some one at home to do this syringing for him. This treatment makes a wonderful improvement even in two or three days; the painful condition of the gums ceases, foul odor of the breath disappears, and the gums take on a healthy appearance. The reasons for this rapid improvement are:

(1) The organisms which cause this condition, fusiform bacilli and spirochætes, are easily washed out if enough force is used in syringing.

(2) Those organisms that are left are killed by the oxygen from the potassium permanganate frequently used, as these germs are anaerobic, and also will not flourish in the absence of necrosed tissue.

(3) The force of the syringing acts as a gum stimulant, and the blood supply is increased.

I have practiced the above treatment now for the last four years with good results, in England and abroad. One finds more cases of this kind in the tropics than in England, but lately they seem to be increasing here.—Albert B. Cocker, in *British Dental Journal.*

---

Roach Ball and Socket Attachment. No one should use attachments of any kind to teeth that do not require crowns or inlays, for the clasp can be used equally successfully without the mutilation. Let us limit our attachments to teeth that require crowns or inlays so that the ball and socket attachment, by reason of the fact that it is a swivel, can be used on inlays and crowns with less leverage, less liability of leverage being exerted on the anchored tooth, and you can reduce the leverage and strain to the anchored tooth with the ball and socket attachments better than any other form of attachment, clasps not excepted. We may use the ball and socket attachment on cases where recent extractions have been made, where we would not dare to use a cast clasp for the reason that if you have a rigid grip on the tooth and a saddle area that is constantly and rapidly changing, you will have a terrific leverage on the tooth, so the ball and socket attachment, if you use the contact spur and take advantage of the range of settling that will obtain on the construction of these cases, you can place these extension saddle cases with the least leverage on the anchored tooth.

If we place that kind of an attachment in the upper jaw and do not include in it this contact spur as an indirect retainer, we will have on appliance that will wiggle up and down like a lamb's tail. In the lower you have gravity, and it is not so necessary, and yet I would rather have my lower cases extension saddle cases, so they will be continuously and surely in place. I don't like these jiggling jobs. In order to have the appliance held securely in place let us put on contact spurs.

Here is the point that I want to make in conjunction with the ball and socket attachments—like the Kelley, or any of those types that have a universal joint: If you are going to put them into the extension saddle construction, to obtain the best results and maximum satisfaction, I think some provision should be made for this retainer, to place the button as near the gingiva as possible and place the tubes so that the button will be in the gingival end of the tube, permitting the settling. This contact spur will hold the plate up at the heel. Now remember that this contact spur is not bearing any particular load. It is only holding just the weight of the plate. The destructive force, the force that does the damage and that is of any consequence, is the force of mastication. That is relieved entirely by the ball and socket so that the strain is exerted upon the saddle. There is no way that I know of that you can attach an extension saddle to a natural tooth and have that tooth freer from strain than you will get with the ball and socket attachment.

I would like to impress upon you, then, the importance of the contact spur and the indispensableness of it in the upper. You can use the contact spur as a means of tightening. If the saddle does not hold up into apposition, by bending the contact spur toward the tooth you are driving the base up against the tissue and it can be continually adjusted in that way. It is a simple way of tightening the appliances if they are made correctly.—*Summary.*

---

DENTAL HEALTH EDUCATION IN NEW YORK. The New York State Department of Education has a definite plan in view, just starting in operation, a plan of what will be probably known as "public health education."

There are many questions to be considered, and we have a big problem on our hands. One problem is the question of time. Another question with which we must deal is the question of finance.

In taking up this subject of health education we must necessarily give dental health education a very important part, because of its very definite and positive relation to the entire body. We are not going to do this in a spasmodic way, by having dentists occasionally go to schools and give lectures, and perhaps come back in three months, or six months, or a year, and give other lectures; we want to undertake it in a systematic manner. We must start with the schoolteachers and give them the necessary education in dental health, so they may impart that knowledge to the children. It has been my privilege to go to each of the normal training schools of the state, and to impart to them some of this knowledge. The child will take up this subject at stated periods, and will be required to pass an examination in dental health, and to secure an average of 75 per cent. The child will be required also to pass an enamination on the condition of its own mouth and secure an average of 75 per cent. I learn that two of the high schools of New York City to-day are requiring that their students shall have good mouth conditions as one of the requirements for graduation.

We will interweave the subject of dental health with the other studies. In spelling we will introduce dental nomenclature. In reading we will have them read dental literature. In arithmetic we can tell them the amount of stress required to masticate certain amounts of food. In zoology and biology we can show the different dental equipments of various animals, and how they are arranged to take care of the peculiar foods of that section of country, and how the habits of the animals are controlled thereby.

We have been establishing dental dispensaries in many of our towns and cities, and it was my pleasure last week to assist in inaugurating the first rural dispensary, in Nassau county, under the auspices of the Junior Red Cross, and they are going to do that work by automobile.

What we have done is entirely inadequate. We have in Rochester, one of our large cities, very adequate dental equipment, but I want to call your attention also to the little town of Chazy, which is situated four or five miles north of Lake Champlain. They have only 500 school children, but they have the best dental equipment that can be secured, and they have a dentist at full time to take care of them. We have a few wonderfully good examples in the small communities, and one large one at Rochester, but we must have them in other cities.—Dr. W. H. Leak, in *Cosmos* for November.

